# Evidence-based medicine (EBM) for undergraduate medical students in Sudan: sources of information, knowledge about terms, skills related to EBM and attitude toward EBM in Sudan

**DOI:** 10.1186/s12909-021-02902-6

**Published:** 2021-09-04

**Authors:** Elfatih A. Hasabo, Walaa Elnaiem, Abrar Y. Ali, Anfal M. Altahir, Elmuiz A. Hsabo, Malaz I. Ibrahim, Dania M. Modathir, Ryan T. Aljaaly, Malaz M. ElSiddig, Sara M. Abdalbagi, Heitham Awadalla

**Affiliations:** 1 Faculty of Medicine, University of Khartoum, Khartoum, Sudan; 2 Department of Urology, Wythenshawe hospital, Manchester University NHS Foundation Trust, Manchester, United Kingdom; 3 Faculty of Medicine, Nile Valley University, Atbara, Sudan; 4 Faculty of Medicine, University of Gezira, Gezira, Sudan; 5 Faculty of Medicine, University of Kassala, Kassala, Sudan; 6 Department of Community Medicine, Faculty of Medicine, University of Khartoum, Khartoum, Sudan

**Keywords:** Evidence-based medicine, Sudan, Attitude, Source of information, Skills

## Abstract

**Background:**

Evidence-based medicine (EBM) is the use of the current best evidence for patient care. Medical students should critically appraise the research evidence to help them during their clinical practice in the future. We conducted this study to assess the skills, terms and attitude toward EBM.

**Method:**

We conducted a cross-sectional study for medical students from governmental universities. Students completed an online validated questionnaire consisting of several sections to assess skills, attitude and knowledge about terms related to EBM. We used a scale ranging from 1(strongly disagree) to 5(strongly agree) for the 11 questions assessing the attitude and a scale ranging from 1(Poor) to 5(advanced) for EBM skills.

**Results:**

A total of 761 medical students with a mean age of 21.97 ± 1.64 participated in the study. 327 (43 %) of them were males. The most commonly used search engines were Google 690 (91 %) and Wikipedia 465 (61 %). Medical books 719 (94 %) and lecture notes 353 (46 %) were the most common sources for health information. The majority of students rated their skills related to EBM as average and below average for all questions (overall = 2.18 ± 0.8). Students rated their skills as poor (31 %) in locating professional literature, as average (34 %) in searching online databases, poor (42 %) in critical appraisal of a scientific publication reporting findings from clinical research and poor (36 %) in Critical appraisal of available scientific literature. Regarding attitude, the overall mean score was 2.83 ± 0.76. There is no significant difference in attitude score between students with or without EBM training (*P* = 0.2).

The terms with the highest understanding were case-control study (45 %) and case report (44 %) for study design. Median (44 %) and sample size (43 %) for statistics. Incidence (46 %) and prevalence (44 %) for epidemiology.

**Conclusions:**

Medical students have a knowledge gap in skills and terms related to EBM and an average attitude towards EBM. The majority of them were using a nonscientific search engine to obtain medical information. There is a need to educate students about the proper steps for getting the scientific literature and EBM skills.

**Supplementary Information:**

The online version contains supplementary material available at 10.1186/s12909-021-02902-6.

## Background

Researchers defined evidence-based medicine (EBM) as “*conscientious, explicit, and judicious use of current best evidence in making decisions about the care of individual patients*” [1]. EBM was focusing on how clinicians can use published literature. Thereafter, the concept was broadened to include the patient-doctor relationship in clinical practice, and integrating patient’s preference with the physician’s experience and the best available research evidence in the decision-making process [2]. Because of the importance of EBM in building clinical practice on a scientific basis, there is a focus on making the source of evidentiary based skills and information accessible to clinicians by developing reliable clinical practice guidelines [2]. EBM practice can be applied in five steps: step one; converting the current clinical scenario into an answerable question using PICOS mnemonic, step two; identifying the best available research evidence by performing a proper literature review across various databases, step three; critical appraisal of the evidence for its validity, impact, and applicability, step four; applying the results of the appraisal with the clinical experience and patient’s values, and step five; evaluating the process and finding ways to improve it in the next time [3]. EBM has improved diagnosis, clinical judgment, and decision making [4]. Additionally, better outcomes were observed in patients who received evidence-based medical care [5]. EBM is of utmost importance in developing countries for its cost-effectiveness and efficient use of healthcare resources [6, 7].

Medical students are the future health care providers. Therefore, there is an increasing emphasis on exposing them to EBM during their pre-clinical and clinical education [8]. Nowadays, EBM has become a core part of the undergraduate medical education curriculum in many countries. Each step was discussed and the students were trained on translating the appraised evidence into clinical practice, this enhances student’s critical thinking and life-long learning [9]. Among the Iranian medical students, only 24.5 % were familiar with the concept of EBM [10]. In a Hungarian study [11]; students reported average skills in identifying patient’s clinical questions, and finding and critically appraising the scientific literature, and poor skills in detecting the knowledge gaps. Less than 10 % of the Hungarian students had advanced EBM skills [11]. Unfortunately, EBM is not adequately implemented in medical curricula for some developing countries [12], including Sudan where most of the medical students' curricula lack EBM tuition. 

The practice of EBM among physicians in Sudan was found to be less than 56.3 % [13]. Most of the Sudanese physicians have not received proper training in EBM [14]. Lack of skills was the main barrier In 57.6 % of those physicians [13]. However, medical students’ awareness, skills, and attitudes towards EBM practice in Sudan are still unknown. With a better understanding of the situation, educational and practical efforts can be developed to implement EBM into our health system. Therefore, this cross-sectional study is the first attempt to provide valuable evidence of medical students’ awareness, skills, and attitudes towards proper EBM practice.

## Methods

### Study design and settings

This cross-sectional study was conducted among undergraduate medical students at ten public universities in Sudan where we included five public universities in Khartoum state and five universities outside Khartoum state in the study between November 2020 to January 2021.

In Khartoum state, the included governmental universities were: Alzaiem Alazhari university, Al-Neelain University, University of Khartoum, Omdurman Islamic university and University of Bahri. Outside Khartoum state. The other five universities were: University of El-imam El-Mahdi in White Nile state, University of Gezira in Gezira state, Red Sea University in Red Sea state, Nile Vally University in River Nile state and University of Kassala in Kassala state. We conducted this cross-sectional study following the STROBE statement for reporting cross-sectional studies [15].

### Participants

We included all undergraduate medical students older than 18 years who were studying in their 2nd year and above at the faculty of medicine in the selected public university. All undergraduate medical students who refused to participate were excluded from the study.

### Instruments used to measure the variables of interest in the study

Data were collected from undergraduate medical students using this questionnaire (Additional file 1) that contained both open and close-ended questions. The questionnaire was distributed online to undergraduate medical students using google form.

The questionnaire consisted of 5 sections of questions: The first part: included 13 questions to assess sociodemographic data of the participating students, which were: age, gender, name of the university, year of study, marital status, attending a course in biostatistics, attending a course in evidence-based medicine, attending a course in research methodology, having a family member in healthcare, the frequency for reading scientific literature, having internet access and having free internet access at home or university. The second part included two questions to assess the most commonly used search engines and sources of information section (one question about the search engines used to obtain medical information and the other about the main sources of health information). The third part: included six questions to measure the skills in evidence-based medicine among undergraduate medical students and consist of a 5-point Likert scale answers in 5 scales from poor {1} to advanced {5}. The Scale was reproduced from Csertő1 et al. 2019 [11]. Cronbach’s alpha in the original paper for the sub-scales was 0.85. The fourth part: included eleven statements of questions to measure attitude toward using EBM in their future work as a health care professional among participating undergraduate medical students. Statements were evaluated by the students using 5 points Likert scales ranging from strongly disagree {1} to strongly agree {5}. The Scale was reproduced from Csertő1 et al. 2019 [11]. Cronbach’s alpha in the original paper for the sub-scales was 0.71. The last part: To assess the knowledge of evidence-based medicine terms related to statistics, epidemiology and study design. Students evaluated their knowledge on a 5-point categorical scale. The five ratings were: {1} I understand and I could explain to others; {2} Some understanding; {3} I do not understand, but would like to understand; {4} I do not understand, but I think it wouldn’t be helpful to me to understand; {5} No idea about this. The Scale was reproduced from McColl et al. [16] and Nejašmić et al. [17].

### Data collection and Sampling

Due to the COVID-19 pandemic, a convenience sampling method was used to acquire the responses from the participants via online distribution of google form during the study period. We recruited medical students from universities using an online google form. Investigators sent the online questionnaire to the online groups of included universities using social media such as Facebook and WhatsApp and telegram to undergraduate medical students. Weekly, Investigators were reminding undergraduate medical students during the study period in their online groups to participate to ensure broader participation in the study among undergraduate medical students.

### Sample size calculation

The sample size for this study was calculated via The following equation n = z^2^P(1-P)/d^2^ [18]. With a 95 % Confidence Interval (CI), 50 % response distribution and 0.05 margin of error, a sample of 384 participants can be considered as a minimal sample to represent the population.

### Statistical analysis

We analyzed and described data using R software version 4.0.2. Continuous data were presented as mean ± SD, and categorical data were presented as numbers (percentage). We used the Kolmogorov-Smirnov test to check the normality of the data. To find a significant difference between groups, we used an independent t-test for normally distributed data and Mann-Whitney U after rejecting the null hypothesis of the Kolmogorov-Smirnov test of normal distribution. We used the Chi-square test or Fisher exact test to find if there was a significant difference between the groups for categorical data. A P-value less than 0.05 is considered significant.

## Results

### Participants’ information

761 Sudanese undergraduate medical students with a mean age of 21.97 ± 1.64 years participated in this cross-sectional study. 327 (43.0 %) of total participants were males, and the most numbers of participants were from the second year (23.3 %) and the third year (31.7 %). One-fourth of participants (26.15 %) received or enrolled in training for evidence-based medicine. Regarding frequency for reading scientific literature, the majority of the students were either never read any scientific literature 193 (25.4 %) or read them monthly or less frequent 389 (51.1 %). Others baseline characteristics for the included medical students were shown in Table 1.

**Table 1:** Baseline characteristics of medical students who completed the online survey in Sudanese universities (*n* = 761)

Variables	Overall, *N* = 761	^EBM Training^		*p*-value^*^
		**Yes**, *N* = 199	**No**,* N* = 562	
**Age (Years)**	21.97 ± 1.64	22.21 ± 1.72	21.89 ± 1.60	0.117
**Gender**				**0.003**
Female	434 (57.0 %)	95 (47.7 %)	339 (60.3 %)	
Male	327 (43.0 %)	104 (52.3 %)	223 (39.7 %)	
**Marital status**				**0.019**
Married	22 (2.9 %)	11 (5.5 %)	11 (2.0 %)	
Single	739 (97.1 %)	188 (94.5 %)	551 (98.0 %)	
**Year in university**				**0.004**
Second	177 (23.3 %)	33 (16.6 %)	144 (25.6 %)	
Third	241 (31.7 %)	81 (40.7 %)	160 (28.5 %)	
Fourth	166 (21.8 %)	34 (17.1 %)	132 (23.5 %)	
Fifth	127 (16.7 %)	36 (18.1 %)	91 (16.2 %)	
Sixth	50 (6.6 %)	15 (7.5 %)	35 (6.2 %)	
**Received or attended any physical or online course in biostatistics (yes)**	300 (39.4 %)	124 (62.3 %)	176 (31.3 %)	**< 0.001**
**Received or attended any physical or online course in Research methodology (Yes)**	382 (50.2 %)	143 (71.9 %)	239 (42.5 %)	**< 0.001**
**Family member (parent, sibling, spouse,.etc)working in health care (Yes)**	427 (56.1 %)	122 (61.3 %)	305 (54.3 %)	0.1
**Frequency of reading scientific literature**:				**< 0.001**
Never	193 (25.4 %)	34 (17.1 %)	159 (28.3 %)	
Daily	49 (6.4 %)	20 (10.1 %)	29 (5.2 %)	
Weekly	130 (17.1 %)	48 (24.1 %)	82 (14.6 %)	
Monthly or less frequent	389 (51.1 %)	97 (48.7 %)	292 (52.0 %)	
**Free internet access at your university or home (Yes)**	315 (41.4 %)	84 (42.2 %)	231 (41.1 %)	0.9
**Internet access (Yes)**	720 (94.6 %)	190 (95.5 %)	530 (94.3 %)	0.7
**Which of the following do you have**:				
Private computer or laptop	454 (59.7 %)	121 (60.8 %)	333 (59.3 %)	0.8
Tablet	145 (19.1 %)	36 (18.1 %)	109 (19.4 %)	0.8
Smart phone	694 (91.2 %)	177 (88.9 %)	517 (92.0 %)	0.2

Data were presented as Mean ± SD and number (percentage)

^*^Statistical tests performed: Independent T-test; chi-square test of independence; Fisher’s exact test

### Search engine and sources of healthcare information retrieval

Among the total medical students who participated in the study, students selected Google search 690 (90.7 %) and Wikipedia 465 (61.1 %) as the most common search engines among medical students. Nearly one third of total participant were using PubMed/Medline 245 (32.2 %) and Medscape 259 (34.0 %). Also, medical students who were enrolled in an EBM training were found to have a significant higher percentage for using PubMed/Medline (41.2 % vs. 29 %; *p* = 0.002), Medscape (44.7 % vs. 30.2 %; *p* < 0.001), Cochrane Library (11.1 % vs. 4.4 %; *p* = 0.002) and Embase (3 % vs. 0.7 %; *p* = 0.024).

Regarding the question “What do you see as the main source of health information? “, nearly all of them 719 (94.5 %) chose medical books as the main source of information, followed by lecture notes 353 (46.4 %). Students who received training in EBM were found selected professional guidelines more than students who didn’t enrol in EBM training (45.2 % vs 34.3 %; *p* = 0.008). More details about the search engines and sources for health information in Table 2.

**Table 2:** Search engines and main sources of health information among Sudanese medical students (*n* = 761)

Variables	Overall, *N* = 761	EBM Training		*p*-value^*^
		**Yes**, *N* = 199	**No**, *N* = 562	
**Search engines used**:				
Google	690 (90.7 %)	175 (87.9 %)	515 (91.6 %)	0.2
Google scholar	243 (31.9 %)	66 (33.2 %)	177 (31.5 %)	0.7
Wikipedia	465 (61.1 %)	119 (59.8 %)	346 (61.6 %)	0.7
PubMed/Medline	245 (32.2 %)	82 (41.2 %)	163 (29.0 %)	**0.002**
Medscape	259 (34.0 %)	89 (44.7 %)	170 (30.2 %)	**< 0.001**
Cochrane Library	47 (6.2 %)	22 (11.1 %)	25 (4.4 %)	**0.002**
Scopus	13 (1.7 %)	6 (3.0 %)	7 (1.2 %)	0.11
Web of science	68 (8.9 %)	18 (9.0 %)	50 (8.9 %)	> 0.9
Embase	10 (1.3 %)	6 (3.0 %)	4 (0.7 %)	**0.024**
Ovid	2 (0.3 %)	1 (0.5 %)	1 (0.2 %)	0.5
Others.	31 (4.1 %)	9 (4.5 %)	22 (3.9 %)	0.9
**Main sources of health information: (You can choose multiple answers)**				
Medical books	719 (94.5 %)	187 (94.0 %)	532 (94.7 %)	0.9
Scientific journals	313 (41.1 %)	83 (41.7 %)	230 (40.9 %)	> 0.9
Electronic media	271 (35.6 %)	66 (33.2 %)	205 (36.5 %)	0.5
Professional guidelines	283 (37.2 %)	90 (45.2 %)	193 (34.3 %)	**0.008**
Leaflets	43 (5.7 %)	10 (5.0 %)	33 (5.9 %)	0.8
Lecture notes	353 (46.4 %)	92 (46.2 %)	261 (46.4 %)	> 0.9
Opinion of health professionals	218 (28.6 %)	57 (28.6 %)	161 (28.6 %)	> 0.9
Others	5 (0.7 %)	0 (0.0 %)	5 (0.9 %)	0.3

Data were presented as Mean ± SD and number (percentage)

^*^Statistical tests performed: Chi-square test of independence; Fisher’s exact test

### Self-reported skills in EBM

The overall rating score of medical students for skills was 2.18 ± 0.80, which is considered a limited experience. The majority of students rating their skill as limited experience in locating professional literature 2.17 ± 1.01 and Identifying patient-relevant clinical questions 2.39 ± 1.07, and the ability for medical students to search online databases was rated as average 2.59 ± 1.11. Also, the remaining skills were rated by students as poor for critical appraisal of scientific papers from clinical research 1.90 ± 0.94 or scientific literature 2.01 ± 0.96 and identifying knowledge gaps in practice 2.04 ± 0.98 (Table 3). Fewer students rated their skills as above average or advanced for EBM skills (Table 3).

**Table 3:** Responses on a 5-point scale to the question: “How would you rate your skills in the following areas?” among Sudanese medical students (*n* = 761)

How would you rate your skills in the following areas?	Poor	Limited experience	Average	Above Average	Advanced	Students with EBM training	Students without EBM training	*P* – Value^*^
Locating professional literature.	234 (30.7 %)	251 (33.0 %)	197 (25.9 %)	68 (8.9 %)	11 (1.4 %)	2.34 ± 1.01	2.11 ± 1.01	**0.004**
Searching online databases.	154 (20.2 %)	192 (25.2 %)	258 (33.9 %)	123 (16.2 %)	34 (4.5 %)	2.76 ± 1.06	2.53 ± 1.13	**0.006**
Critical appraisal of a scientific publication reporting findings from clinical research.	319 (41.9 %)	253 (33.2 %)	147 (19.3 %)	32 (4.2 %)	10 (1.3 %)	2.03 ± 0.98	1.85 ± 0.93	**0.025**
Identifying knowledge gaps in practice (fields where not enough scientific literature is available to answer specific clinical question).	271 (35.6 %)	255 (33.5 %)	176 (23.1 %)	49 (6.4 %)	10 (1.3 %)	2.23 ± 1.00	1.98 ± 0.97	**0.002**
Critical appraisal of available scientific literature.	273 (35.9 %)	265 (34.8 %)	173 (22.7 %)	39 (5.1 %)	11 (1.4 %)	2.20 ± 0.97	1.95 ± 0.95	**< 0.001**
Identifying patient-relevant clinical questions.	178 (23.4 %)	254 (33.4 %)	210 (27.6 %)	96 (12.6 %)	23 (3.0 %)	2.56 ± 1.03	2.32 ± 1.07	**0.004**
**Overall score**						2.35 ± 0.79	2.12 ± 0.80	**< 0.001**

Data were presented as Mean ± SD and number (percentage)

^*^Statistical tests performed: Mann-Whitney U test

Medical students who received or participated in EBM training rated all the items for EBM skills significantly higher than those who didn’t receive the EBM course (Table 3).

### Attitudes of medical students towards using EBM in health care practice

The overall attitude score for medical students was 2.83 ± 0.76. Although, the majority of them agreed that evidence-based medicine (EBM) is essential for the practical work of physicians 3.92 ± 1.17 (Table 4). But we didn’t find any difference in the attitude score for the ten items of attitude out of 11 between medical students who received or didn’t receive the EBM course (Table 4). The only significant difference in attitude score was found for the statement “Textbooks are the most optimal source of information, when a question regarding the care of patients should be answered” (*p =* 0.016), and it was higher for students who received EBM course (Table 4).

**Table 4:** Response frequency and means of ratings to the question: “On a scale ranging from ‘strongly disagree’ to ‘strongly agree’ how would you rate your opinion about the following statements?” among Sudanese medical students (*n* = 761)

Questions	Strongly disagree	Disagree	Neutral	Agree	Strongly agree	Students with EBM training	Students without EBM training	*P* – Value^*^
	1	2	3	4	5			
Evidence based medicine (EBM) is important for the practical work of physicians	54 (7.1 %)	33 (4.3 %)	129 (17.0 %)	248 (32.6 %)	297 (39.0 %)	3.95 ± 1.12	3.84 ± 1.31	0.9
During my studies, I would like to improve my skills in applying EBM during my practical work as a medical professional	52 (6.8 %)	41 (5.4 %)	134 (17.6 %)	239 (31.4 %)	295 (38.8 %)	3.92 ± 1.14	3.83 ± 1.27	0.7
EBM is important for patients to receive the optimal treatment	46 (6.0 %)	55 (7.2 %)	147 (19.3 %)	241 (31.7 %)	272 (35.7 %)	3.87 ± 1.11	3.74 ± 1.32	0.5
EBM facilitates decisions about individual patient’s care	46 (6.0 %)	48 (6.3 %)	181 (23.8 %)	257 (33.8 %)	229 (30.1 %)	3.78 ± 1.08	3.68 ± 1.26	0.6
EBM considers the personal expertise of physicians	61 (8.0 %)	91 (12.0 %)	268 (35.2 %)	239 (31.4 %)	102 (13.4 %)	3.32 ± 1.04	3.25 ± 1.24	0.5
EBM considers views and preferences of patients regarding their own therapy	62 (8.1 %)	112 (14.7 %)	282 (37.1 %)	205 (26.9 %)	100 (13.1 %)	3.26 ± 1.07	3.12 ± 1.20	0.088
It is important to incorporate research results into healthcare practice	58 (7.6 %)	51 (6.7 %)	165 (21.7 %)	237 (31.1 %)	250 (32.9 %)	3.78 ± 1.13	3.65 ± 1.38	0.7
All types of studies are of equal value	186 (24.4 %)	285 (37.5 %)	178 (23.4 %)	81 (10.6 %)	31 (4.1 %)	2.36 ± 1.09	2.22 ± 1.03	0.1
EBM means an unrealistic burden to health care professionals in the daily routine patient care	165 (21.7 %)	228 (30.0 %)	242 (31.8 %)	100 (13.1 %)	26 (3.4 %)	2.50 ± 1.05	2.38 ± 1.13	0.15
Textbooks are the most optimal source of information, when a question regarding the care of patients should be answered	97 (12.7 %)	196 (25.8 %)	235 (30.9 %)	179 (23.5 %)	54 (7.1 %)	2.92 ± 1.11	2.72 ± 1.16	**0.016**
As a future healthcare practitioner, I find life-long learning as vital	71 (9.3 %)	60 (7.9 %)	167 (21.9 %)	214 (28.1 %)	249 (32.7 %)	3.67 ± 1.23	3.66 ± 1.35	0.7
**Mean Overall score**						2.88 ± 0.83	2.81 ± 0.73	0.2

Data were presented as Mean ± SD and number (percentage)

^*^Statistical tests performed: Mann-Whitney U test

### Knowledge of terms related to EBM

There was a massive difference in understanding EBM terms. The most known and understandable term related to study design terms was case-control study 342 (44.9 %), followed by case report 335 (44.0 %). Unfortunately, meta-analysis 115 (15.1 %) and systematic review 200 (26.3 %) were the least known terms related to study design (Table 5).

**Table. 5:** Self-reported understanding of evidence-based healthcare-related terms among Sudanese medical students (*n* = 761)

	**I understand and I could explain to others.**	**Some understanding.**	**Do not understand, but would like to understand.**	**Do not understand, but I think, it wouldn’t be helpful to me to understand**	**No idea about this**
**Terms related to study design**:					
Case report	335 (44.0 %)	279 (36.7 %)	110 (14.5 %)	11 (1.4 %)	26 (3.4 %)
Cohort study	286 (37.6 %)	278 (36.5 %)	135 (17.7 %)	13 (1.7 %)	49 (6.4 %)
Randomized Controlled clinical trial	202 (26.5 %)	276 (36.3 %)	203 (26.7 %)	24 (3.2 %)	56 (7.4 %)
Meta-analysis	115 (15.1 %)	275 (36.1 %)	277 (36.4 %)	28 (3.7 %)	66 (8.7 %)
Systematic review	200 (26.3 %)	280 (36.8 %)	212 (27.9 %)	19 (2.5 %)	50 (6.6 %)
Cross-sectional study	315 (41.4 %)	244 (32.1 %)	153 (20.1 %)	16 (2.1 %)	33 (4.3 %)
Case–control study	342 (44.9 %)	235 (30.9 %)	139 (18.3 %)	14 (1.8 %)	31 (4.1 %)
**Terms related to statistics**:					
Confidence interval	146 (19.2 %)	284 (37.3 %)	236 (31.0 %)	20 (2.6 %)	75 (9.9 %)
Sample size	330 (43.4 %)	250 (32.9 %)	122 (16.0 %)	17 (2.2 %)	42 (5.5 %)
Mode	292 (38.4 %)	212 (27.9 %)	176 (23.1 %)	18 (2.4 %)	63 (8.3 %)
Median	334 (43.9 %)	203 (26.7 %)	155 (20.4 %)	13 (1.7 %)	56 (7.4 %)
Interquartile range (IQR)	146 (19.2 %)	239 (31.4 %)	263 (34.6 %)	31 (4.1 %)	82 (10.8 %)
Standard deviation (SD)	248 (32.6 %)	250 (32.9 %)	174 (22.9 %)	28 (3.7 %)	61 (8.0 %)
Precision and accuracy	145 (19.1 %)	245 (32.2 %)	269 (35.3 %)	27 (3.5 %)	75 (9.9 %)
Representative sample	213 (28.0 %)	260 (34.2 %)	201 (26.4 %)	23 (3.0 %)	64 (8.4 %)
Test power	103 (13.5 %)	233 (30.6 %)	305 (40.1 %)	29 (3.8 %)	91 (12.0 %)
*P*-value	146 (19.2 %)	252 (33.1 %)	241 (31.7 %)	32 (4.2 %)	90 (11.8 %)
Type I and type II errors	114 (15.0 %)	247 (32.5 %)	281 (36.9 %)	26 (3.4 %)	93 (12.2 %)
**Terms related to Epidemiology**:					
Relative risk	248 (32.6 %)	275 (36.1 %)	170 (22.3 %)	15 (2.0 %)	53 (7.0 %)
Absolute risk	223 (29.3 %)	273 (35.9 %)	197 (25.9 %)	19 (2.5 %)	49 (6.4 %)
Odds ratio	175 (23.0 %)	269 (35.3 %)	227 (29.8 %)	22 (2.9 %)	68 (8.9 %)
NNT (number needed to treat)	118 (15.5 %)	223 (29.3 %)	302 (39.7 %)	23 (3.0 %)	95 (12.5 %)
Sensitivity of a diagnostic test	238 (31.3 %)	233 (30.6 %)	213 (28.0 %)	23 (3.0 %)	54 (7.1 %)
Specificity of a diagnostic test	232 (30.5 %)	235 (30.9 %)	221 (29.0 %)	18 (2.4 %)	55 (7.2 %)
Heterogeneity	138 (18.1 %)	213 (28.0 %)	296 (38.9 %)	34 (4.5 %)	80 (10.5 %)
Publication bias	146 (19.2 %)	227 (29.8 %)	276 (36.3 %)	26 (3.4 %)	86 (11.3 %)
Lost to follow-up	187 (24.6 %)	238 (31.3 %)	224 (29.4 %)	29 (3.8 %)	83 (10.9 %)
Randomization	270 (35.5 %)	247 (32.5 %)	164 (21.6 %)	21 (2.8 %)	59 (7.8 %)
Intention-to-treat analysis	115 (15.1 %)	211 (27.7 %)	310 (40.7 %)	24 (3.2 %)	101 (13.3 %)
Prevalence	332 (43.6 %)	241 (31.7 %)	123 (16.2 %)	20 (2.6 %)	45 (5.9 %)
Incidence	347 (45.6 %)	232 (30.5 %)	118 (15.5 %)	23 (3.0 %)	41 (5.4 %)
Positive predictive value	163 (21.4 %)	231 (30.4 %)	260 (34.2 %)	28 (3.7 %)	79 (10.4 %)
Hierarchy of evidence	117 (15.4 %)	200 (26.3 %)	315 (41.4 %)	28 (3.7 %)	101 (13.3 %)
Clinical effectiveness	146 (19.2 %)	246 (32.3 %)	274 (36.0 %)	27 (3.5 %)	68 (8.9 %)
Practical guideline	160 (21.0 %)	258 (33.9 %)	255 (33.5 %)	21 (2.8 %)	67 (8.8 %)
Evidence-based medicine	230 (30.2 %)	279 (36.7 %)	186 (24.4 %)	23 (3.0 %)	43 (5.7 %)

Data were presented as number (percentage)

Regarding terms related to statistics, most of the students showed less understanding for this domain, and median 334 (43.9 %) and sample size 330 (43.4 %) were the most known understandable term (Table 5).

Regarding terms related to epidemiology, students identified incidence 347 (45.6 %) and prevalence 332 (43.6 %) as the most known terms for them, and intention-to-treat analysis 115 (15.1 %) as the least known term (Table 5).

## Discussion

This cross-sectional study was designed to map the attitudes, knowledge and skills related to EBM among medical students in Sudan. The attitude towards EBM was generally positive, a finding that was demonstrated in other studies as well [17, 19–24]. However, our study revealed that lacking prior training in EBM did not result in an inferior perception of EBM as one might expect. In fact, the only difference observed between the two groups regarding the attitude towards EBM was the gravitation towards textbooks as key source of information guiding patient care. We noticed that students with EBM training were more likely to consult a textbook when attempting to answer a clinical question. Furthermore, the majority of the students were neutral regarding the statement ‘EBM considers views and preferences of patients regarding their own therapy’.

On analyzing the students’ responses to the questions assessing the level of understanding of EBM-related terms, we noticed considerable differences in their comprehension of the various EBM-related terms. The majority of students felt confident with their knowledge of the concept of case report (80.7 %), meta-analysis seemed to be the weakest area among the terms related to study design (51.2 %). Regarding the statistical terms, we found that the ’sample size’ was the most understood term (76.3 %), followed by the ‘median’ (70.6 %). Almost half of the students could not explain the advanced terms like Type I and type II errors, ‘test power’, and interquartile range. This finding could be partly explained by the fact that most of the participating universities start teaching their students advanced statistical and epidemiological topics earlier during their study.

Generally, most of the students involved in the study declared having limited experience in the skills pertaining to applying EBM. This is particularly true when it comes to the ability to critique a scientific publication where the two groups saw the worst performance on average. As expected, students with EBM training scored higher than their counterparts who did not receive any training. It is also worth mentioning that EBM-trained students reported significantly better skills in locating professional literature, searching online databases, critiquing a scientific paper, identifying knowledge gap in practice and pinpointing patient-specific clinical questions. Unfortunately, most of the Sudanese medical students had less EBM skills than Hungarian medical students who show an average EBM skill [11].

Two similar studies were identified from the literature: a survey with 1080 participants in Hungary [11] and a cross-sectional study conducted in Iran [10]. Across all studies, there was generally a positive attitude toward EBM. Similarly, both studies demonstrated limited formal training in EBM among students, low utilization of the advanced online EBM resources (like Cochrane) and poor familiarity with the various EBM-related terms. The data from F. Ghahremanfard et al. [10] suggested that students depend on textbooks and expert opinions when seeking clinical information. This is different from our study, which showed that medical journals and lecture notes were the major sources of information. Csertő M, et al. [11] concluded that students who had received training in EBM tended to be significantly better at critical appraisal and knowledge gaps identification, which is similar to our finding.

We identified another questionnaire-based survey [25] performed among medical students at the Faculty of Medicine, University of Damascus (Syria) where 50 students were given an EBM course and an identical set of test questionnaires were distributed before and after completing the course. Unlike our study, the percentage of students who had received EBM training beforehand was noticeably higher in their study (56 % vs. 26.15 %). That is being said, a lot of similarities had risen upon comparing our results with their baseline (pre-course) findings. For instance, it appears that the majority of the students had conducted an online search for literature infrequently (< once per week) (68 % vs. 76.5 %), with almost half of the students found it difficult to search for the literature online (47.9 % vs. 45.4 %) and only limited number of them felt competent at critically appraising a scientific paper (34 % vs. 24.8 %). Moreover, Google was the most used search engine to search the literature in both studies (46 % vs. 90.7 %), a finding that can be explained -in part- by the lack of institutional access to subscription journals. When it comes to the attitudes toward EBM, the vast majority of students were keen to receive professional training in EBM (98 % vs. 87.8 %).

Regarding terms related to EBM, Incidence rate (37.8 %) and Publication bias (21.7 %) were found to be the most common understandable terms among Iranian medical students [10]. In another study, sample size (65.09 %) and case study (59.07 %) were the most common understandable terms among Hungarian students [11]. These findings are different from our study, which identified incidence (45.6 %) and case-control study (44.9 %) as the most understandable terms among Sudanese medical students. On the other hand, other studies showed a low awareness of extracting journals, review publications, and databases relevant to evidence-based medicine among general practitioners [19–21, 23].

Previously, tools such as Fresno tool [26] and assessing competency in EBM (ACE) tool [27] were used to evaluate EBM steps and skills. The ACE tool consists of 15 items that assess the 5- steps of EBM which was validated among medical trainees [27], and the Fresno tool for assessing EBM skills [26]. Fresno tool consists of 12 items and it was validated and used in a previous study among pharmacy students [28]. Both tools assess the ability to apply steps of EBM and skills by giving an example and this is different from our study.

As far as we know, this is the first study that compared attitudes, knowledge and skills between Sudanese medical students who participated in an EBM training course and those who did not. One of the strength points of our study is the large sample size and multiple included universities, which could dampen the impact of response bias on the study findings. Because of this broad coverage, the results of this study are likely to be generalizable to the Sudanese medical students, and this study can serve as a starting point for future studies in countries with similar characteristics. Our results also highlight the importance of establishing focused, unified courses in EBM across all Sudanese universities and reiterating the need to apply EBM to individual patient care.

Despite the strength of our study, we encountered several limitations. Firstly, the responses recorded in the survey are self-reported and hence subjective. Therefore, they may not reflect the actual knowledge and the ability of an individual to apply EBM into their practice. Moreover, we did not ascertain the level of EBM training an individual had received; the exact number of hours engaged or the type of session delivered -whether face-to-face or online-. Lastly, our study sample might not have been representative of all Sudanese medical students, mainly because of COVID-19 pandemic in addition to financial difficulties that restricted some students' access to the internet. Therefore, some students were not able to participate in the study.

## Conclusions

Medical students have a knowledge gap in skills and terms related to EBM and an average attitude towards using EBM in health care practice. The majority were using a nonscientific search engine to obtain medical information due to the lack of institutional access in universities. They get medical information from medical books and lecture notes. There is a need to educate students about the proper steps for obtaining scientific literature.

To fill this gap in EBM among medical students, further interventional studies and training in EBM are required to improve EBM knowledge among medical students. Also, University staff should focus on secondary research such as systematic review and meta-analysis; because most of the participants don’t know them. To apply this knowledge, practical sessions for improving the skills of EBM should be included in the curriculum to enhance their skills. Lastly, the Ministry of higher education and universities should inform medical students about the importance of EBM and establish scientific meetings to motivate students to be involved in research and increase their knowledge about EBM.

## Supplementary Information

**Additional file 1.**

## Data Availability

The datasets used and/or analyzed during the current study are available from the corresponding author on reasonable request.
