# Supplementary material for: Evidence-based medicine (EBM) for undergraduate medical students in Sudan: sources of information, knowledge about terms, skills related to EBM and attitude toward EBM in Sudan
Source: BMC Med Educ. 2021 Sep 4;21:470. doi: 10.1186/s12909-021-02902-6 (PMC8417679; doi:10.1186/s12909-021-02902-6)
Supplement: Supplementary file 1 — Additional file 1. [file 12909_2021_2902_MOESM1_ESM.docx]

**University of Khartoum**

**Faculty of Medicine**

**Department of Community Medicine**

Questionnaire on:

**Evidence-based medicine (EBM) for undergraduate medical students in Sudan: sources of information, knowledge about terms, skills related to EBM and attitude toward EBM in Sudan.**

- We are a group of researchers conducting a study about the "**Sources of information, skills, attitude and knowledge about evidence-based medicine(EBM) among medical students at governmental universities in Sudan**."
- All medical students from 2^nd^ year till 6^th^ year are invited to fill this questionnaire.
- It will take between 2 -4 minutes to fill this questionnaire.
- Kindly answer all the questions by putting the answer's number in the corresponding square.
- Participation in this research is voluntary, and all information is anonymous and will be used only for research purposes.
- The participant can quit the research at any time.
- By agreeing to complete this form, you permit us to use the data in our research project.

|  |  |  |
| --- | --- | --- |

Questionnaire no:

Do you agree to participate in this study?

1. Yes
2. No

**Socio-demographic information: -**

**In this section, we will gather Socio-demographic about the student**.

- 1. What is your Age?
  2. What is your gender?
     1. Male 2. Female
  3. What is the name of your University?
     1. University of Khartoum.
     2. Bahri University.
     3. Omdurman Islamic University.
     4. Alneelain University.
     5. Alzaeem AlAzhari University.
     6. Red sea University.
     7. Kassala University.
     8. Nile Valley University.
     9. University of Gezira.
     10. University of El-imam El-Mahdi
  4. What is your University level?
     1. Second-year
     2. Third-year.
     3. Fourth-year
     4. Fifth-year.
     5. Sixth year.
  5. What is your Marital status?
     1. Single
     2. Married
     3. Widow
     4. Divorced
  6. Did you receive or attend any physical or online course in biostatistics?
     1. Yes
     2. No
  7. Did you receive or attend any physical or online course in Evidence-based

medicine?

- - 1. Yes
    2. No
  1. Did you receive or attend any physical or online course in research

methodology?

- - 1. Yes
    2. No
  1. Do you have near family members (parent, sibling, spouse,….etc) working

in health care:

- - 1. Yes
    2. No
  1. How often do you read scientific literature?

1. Daily

2. Weekly

3. Monthly or less frequent

4. Never

- 1. Do you have free internet access at your University or home?

1. Yes

2. No

- 1. Do you have internet access?

1. Yes

2. No

- 1. Which of the following do you have? **(multiple answer questions)**

1. Private computer or laptop

2. Tablet

3. Smartphone

4. Never

**Source of information:**

1. Which of the following search engines have you already used to obtain medical / health information? **(You can choose multiple answers)**

1. Google

2. Google Scholar

3. Wikipedia

4. PubMed/Medline

5. Medscape

6. Cochrane Library

7. Scopus

8. Web of science

9. Embase

10. Ovid

1. What do you see as the main source of health information? **(You can choose**

**multiple answers)**

1. Medical books

2. Scientific journals

3. Electronic media

4. Professional guidelines

5. Leaflets

6. Lecture notes

7. Opinion of health professionals

**Skills in EBM:**

How would you rate your skills in the following areas? (You can choose either: Poor, Limited experience, Average, Above average, Advanced)

| **Questions** | **Poor** | **Limited experience** | **Average** | **Above average** | **Advanced** |
| --- | --- | --- | --- | --- | --- |
| 1. Locating professional literature | O | O | O | O | O |
| 2. Searching online databases | O | O | O | O | O |
| 3. Critical appraisal of a scientific publication reporting findings from clinical research | O | O | O | O | O |
| 4.Identifying knowledge gaps in practice (fields where not enough scientific literature is available to answer a specific clinical question) | O | O | O | O | O |
| 5. Critical appraisal of available scientific literature | O | O | O | O | O |
| 6.Identifying patient-relevant clinical questions. | O | O | O | O | O |

**Attitude toward EBM:**

How much do you agree with the following statements? (You can choose either: Strongly disagree, Disagree, Neutral, Agree or Strongly Agree)

| **Questions** | **Strongly disagree** | **Disagree** | **Neutral** | **Agree** | **Strongly agree** |
| --- | --- | --- | --- | --- | --- |
| 1. Evidence-based medicine (EBM) is important for the practical work of physicians. | O | O | O | O | O |
| 2. During my studies, I would like to improve my skills in applying EBM during my practical work as a medical professional. | O | O | O | O | O |
| 3. EBM is important for patients to receive the optimal treatment. | O | O | O | O | O |
| 4. EBM facilitates decisions about individual patient's care | O | O | O | O | O |
| 5. EBM considers the personal expertise of physicians | O | O | O | O | O |
| 6. EBM considers views and preferences of patients regarding their own therapy | O | O | O | O | O |
| 7. It is important to incorporate research results into healthcare practice | O | O | O | O | O |
| 8. All types of studies are of equal value | O | O | O | O | O |
| 9. EBM means an unrealistic burden to health care professionals in the daily routine patient care | O | O | O | O | O |
| 10. Textbooks are the most optimal source of information, when a question regarding the care of patients should be answered | O | O | O | O | O |
| 11.As a future healthcare practitioner, I find life-long learning as vital | O | O | O | O | O |

**Knowledge of EBM-related terms:**

How familiar are you with the following concepts? (You can choose only one answer for each term)

|  | **I understand and I could explain to others.** | **Some understanding.** | **Do not understand, but would like to understand.** | **Do not understand, but I think, it wouldn't be helpful to me to understand** | **No idea about this** |
| --- | --- | --- | --- | --- | --- |
| **1- Terms related to study design:** |  |  |  |  |  |
| 1. Case report | O | O | O | O | O |
| 2. Cohort study | O | O | O | O | O |
| 3. Randomized Controlled clinical trial | O | O | O | O | O |
| 4. Meta-analysis | O | O | O | O | O |
| 5. Systematic review | O | O | O | O | O |
| 6. Cross-sectional study | O | O | O | O | O |
| 7. Case-control study | O | O | O | O | O |
| **2- Terms related to statistics:** | | | | | |
| 1. Confidence interval | O | O | O | O | O |
| 2. Sample size | O | O | O | O | O |
| 3. Mode | O | O | O | O | O |
| 4. Median | O | O | O | O | O |
| 5. Interquartile range (IQR) | O | O | O | O | O |
| 6. Standard deviation (SD) | O | O | O | O | O |
| 7. Precision and accuracy | O | O | O | O | O |
| 8.Representative sample | O | O | O | O | O |
| 9. Test power | O | O | O | O | O |
| 10. P-value | O | O | O | O | O |
| 11. Type I and type II errors | O | O | O | O | O |
| **3- Terms related to Epidemiology:** | | | | | |
| 1. Relative risk | O | O | O | O | O |
| 2. Absolute risk | O | O | O | O | O |
| 3. Odds ratio | O | O | O | O | O |
| 4. NNT (number needed to treat) | O | O | O | O | O |
| 5. Sensitivity of a diagnostic test | O | O | O | O | O |
| 6. Specificity of a diagnostic test | O | O | O | O | O |
| 7. Heterogeneity | O | O | O | O | O |
| 8. Publication bias | O | O | O | O | O |
| 9. Lost to follow-up | O | O | O | O | O |
| 10. Randomization | O | O | O | O | O |
| 11. Intention-to-treat analysis | O | O | O | O | O |
| 12.Prevalence | O | O | O | O | O |
| 13. Incidence | O | O | O | O | O |
| 14. Positive predictive value | O | O | O | O | O |
| 15. Hierarchy of Evidence | O | O | O | O | O |
| 16. Clinical effectiveness | O | O | O | O | O |
| 17. Practical guideline | O | O | O | O | O |
| 18. Evidence-based medicine | O | O | O | O | O |
